# Olfactory Ecto-Mesenchymal Stem Cells in Modeling and Treating Alzheimer’s Disease

**DOI:** 10.3390/ijms25158492

**Published:** 2024-08-03

**Authors:** Hongmin Wang, Abena Dwamena

**Affiliations:** Department of Pharmacology and Neuroscience, Garrison Institute on Aging, Center of Excellence for Translational Neuroscience and Therapeutics, Texas Tech University Health Science Center, Lubbock, TX 79424, USA; adwamena@ttuhsc.edu

**Keywords:** Alzheimer’s disease, olfactory, mesenchymal stem cell, therapeutic, model, differentiation, neuron, glia, transplantation, exosome

## Abstract

Alzheimer’s disease (AD) is a condition in the brain that is marked by a gradual and ongoing reduction in memory, thought, and the ability to perform simple tasks. AD has a poor prognosis but no cure yet. Therefore, the need for novel models to study its pathogenesis and therapeutic strategies is evident, as the brain poorly recovers after injury and neurodegenerative diseases and can neither replace dead neurons nor reinnervate target structures. Recently, mesenchymal stem cells (MSCs), particularly those from the human olfactory mucous membrane referred to as the olfactory ecto-MSCs (OE-MSCs), have emerged as a potential avenue to explore in modeling AD and developing therapeutics for the disease due to their lifelong regeneration potency and facile accessibility. This review provides a comprehensive summary of the current literature on isolating OE-MSCs and delves into whether they could be reliable models for studying AD pathogenesis. It also explores whether healthy individual-derived OE-MSCs could be therapeutic agents for the disease. Despite being a promising tool in modeling and developing therapies for AD, some significant issues remain, which are also discussed in the review.

## 1. Introduction

The known pathology of Alzheimer’s disease (AD) involves amyloid beta (Aβ) plaques outside neuronal cells and intraneuronal build-up of tangles of hyperphosphorylated tau, leading to the death of brain cells and the loss of neuronal connections. It is estimated that about seven million Americans who are 65 and above are living with AD, which is projected to rise by 2060 to nearly 14 million [1]. AD was the fifth-leading cause of death among people aged 65 and older in 2021. After two decades of efforts, recent progress has led to two anti-amyloid monoclonal antibodies approved by the US Food and Drug Administration to treat early-stage AD: aducanumab and lecanemab [2]. However, both have significant side effects, and clinical trials have been terminated for aducanumab [3].

The central nervous system (CNS) cannot recover following injury or neurodegenerative diseases [4]. These pathological conditions can lead to substantial and continuous neuronal loss and axonal degeneration, typically with limited regeneration, affecting their ability to reinnervate target structures to perform essential functions properly. Some studies have directed their attention to protective interventions in the affected part of the brain to enhance recovery and limit brain lesions. However, it is difficult for the brain to increase plasticity and improve after injury [5]. Presently, there are no treatments to protect or replace affected CNS tissue. Given this, there is a demand for better therapeutic strategies to facilitate restoring neurological function in the CNS. This review aims to contribute to this urgent need by exploring the feasibility of olfactory ecto-mesenchymal stem cells (OE-MSCs) as a promising agent for treating AD and an effective model to study the disease.

Using stem cells as therapies has gained attention as a viable tool to improve CNS dysfunction outcomes [6,7,8]. These unspecialized cells in the body can transform into many diverse cell types in early life as one grows. There has been growing interest in stem cell therapies for their application in advanced regenerative medicine since they can repair and regenerate into specialized types of cells when an appropriate condition is provided [9,10]. Stem cells can be grouped into embryonic, induced pluripotent, and adult (somatic) stem cells [11]. For research, adult stem cells are preferred to avoid ethical issues using cells derived from the embryo and the potential for induced pluripotent stem cells to form tumors [12,13,14]. Among the adult stem cells, a unique subpopulation known as MSCs, of stromal origin can regenerate and transform into multilineage cells despite their high heterogeneity [15]. MSCs can be derived from the bone marrow, umbilical cord, menstrual blood, adipose tissue, skeletal muscle, dental pulp, dermal tissue, and olfactory mucosa [16,17]. While all these sources have their merits, OE-MSCs localized in the nasal cavity have been discovered as a potent source of neural stem cells. Their ease of accessibility and lifelong potency make them highly suitable for intensive examination and therapeutic exploitation in AD and other neurodegenerative diseases [18,19,20,21,22]. 

**Figure 1:**
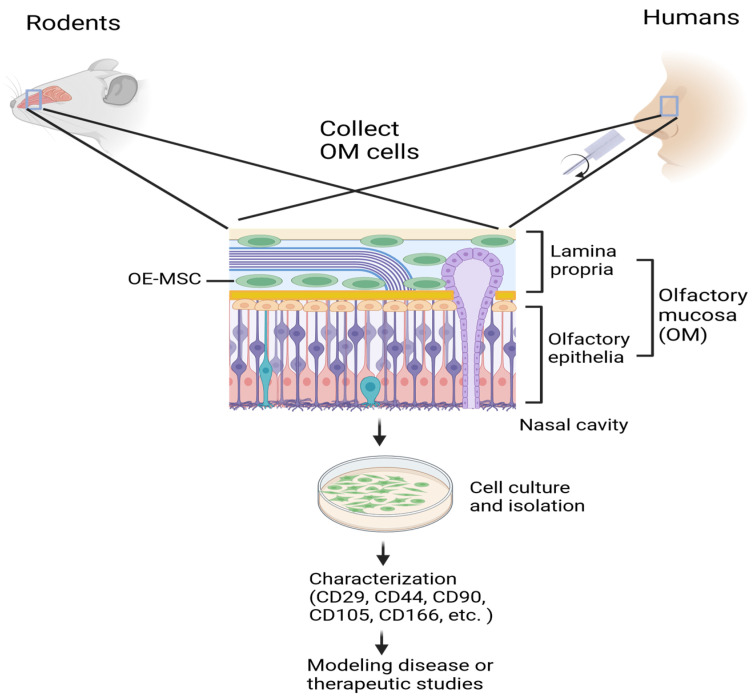
Isolation and culture of OE-MSCs. OE-MSCs can be isolated from rodent or human olfactory mucosa and cultured in a petri dish before being characterized and used for modeling and treating AD.

OE-MSCs are a large group of stem cells found in the olfactory mucous membrane, in the lamina propria region, which can regenerate the olfactory epithelium (Figure 1). They have shown promise in understanding the pathogenesis of AD and developing potential therapeutic strategies, making them a valuable tool in the fight against this devastating disease [23]. Increasing data have suggested that AD patients-derived OE-MSCs may be an ideal model to study the pathogenesis of the disease [24,25]. This potential, coupled with the facile accessibility of healthy-derived OE-MSCs, offers a hopeful outlook for developing therapeutics for various neurodegenerative diseases. Using OE-MSCs could overcome many obstacles associated with using other sources of stem cells, including human embryonic stem cells, and pave the way for significant advancements in AD research.

In this review, we first evaluated methods successfully used by other researchers to collect, isolate, and culture OE-MSCs from rodents and humans to promote interest in their use in research. We then assessed how to differentiate OE-MSCs into neural cells for different studies. Then, we summarized the current literature on using healthy individual-derived OE-MSCs to treat AD. Finally, we discussed the future directions and challenges in using the cells to model and treat AD. 

## 2. Isolation and Culture of OE-MSCs from Rodents and Humans

### 2.1. Isolation and Culture of OE-MSCs from Rodents 

It is noted that the Mesenchymal and Tissue Stem Cell Committee of the International Society for Cellular Therapy proposes the following minimal criteria to define MSC: (1) plastic adherence when maintained in standard culture conditions, (2) expression of CD105, CD73, and CD90, (3) lack of expression of CD45, CD34, CD14 or CD11b, CD79α or CD19, and HLA-DR surface molecules, and (4) ability to spontaneously differentiate into osteoblasts, adipocytes, and chondroblasts in vitro [26].

In order to extract and define OE-MSCs derived from the olfactory bulbs of rodents, some early work initially tried rats and rabbits due to the high tissue yield compared to mice (Figure 1). For instance, culture of the olfactory bulb tissue isolated from adult rats (6–8 weeks) in plastic plates in a medium containing DMEM/F-12 (Dulbecco’s Modified Eagle Medium/Nutrient Mixture F-12) supplemented with 10% fetal bovine serum (FBS), L-glutamine, and antibiotics resulted in many spindle-shaped, fibroblast-like cells after eight hours following the culture [27]. Further characterization of these fibroblast-like cells suggests that they are OE-MSCs. Because the F-12 medium enhances cell growth rate, it has been consistently used in the preparation and propagation of OE-MSC by different research groups [28]. The culture mediums were changed every 2–3 days, and after one week, the cells were subjected to subcultures [29]. The advantage of this approach is that it can result in a high yield of cells and its disadvantage is potential contamination with other types of cells (Table 1). However, these non-MSCs will become fewer and fewer with repeat passages in the culture condition, due to their relatively low proliferation rate compared to the MSCs. Moreover, as fibroblast-like cells adhere to glass plates within an hour, and most astrocytes adhere within 36 h, a medium change after 18 h could lead to 70% cells with fibroblast-like OE-MSCs and 30% with other types of cells [30]. In order to obtain a higher yield of fibroblast-like OE-MSCs with higher purity, a modified protocol was tested by changing the medium after 8 h, and was incubated for an additional week until 70–80% confluence was reached [27]. Adherent cells exhibited a morphology of spindles in the plates on which they were cultured. This protocol obtained relatively pure fibroblast-like cells confirmed to be neither astrocytes nor ensheathing cells through immunocytochemical staining. The fibroblast-like cells also showed normal proliferative characteristics. They could differentiate into lineages that expressed CD29 and CD90 (Thy1.1) on their surfaces but failed to express CD31, CD34, and CD45, further supporting that these cells are indeed MSCs [27]. Moreover, these cells demonstrated pluripotency: after being exposed to differentiation conditions, they became osteoblasts, adipocytes, and chondrogenic lineages of the cell. This work revealed that olfactory bulb tissue is a reliable source of MSCs, and this isolation method produces a better yield with less contamination. Therefore, the advantage of this differential adhesion approach in isolation of OE-MSCs is that it could lead to the relatively high purity of OE-MSCs. Its disadvantage is the relatively low yield of the cells due to some OE-MSCs being taken away before tightly adhered to the culture plate surface (Table 1).

Other authors also performed similar work using olfactory bulbs of either neonatal or adult rats [23]. The cells exhibited fibroblast morphology with the expression of CD29, CD44, CD90, CD105, and CD166 but failed CD34 and CD45, indicative of the characteristics of MSCs [31]. Intriguingly, they differentiated into myocardial-like cells when grown together with rat cardiomyocytes, neuregulin-1, basic fibroblast growth factor, and forskolin [31], indicating pluripotency of the cells. 

In addition to rats and rabbits, other mammals, including mice, sheep, dogs, horses, gray mouse lemur, and macaque, were also utilized to isolate and characterize OE-MSCs [32]. Instead of euthanasia of donor animals, except mice, biopsies of olfactory mucosa were performed in the donor animals. In cell cultures, the cells isolated from different mammals shared similar morphological features: fibroblast-like morphology, nestin, CD44, and CD73 expression, as well as high proliferation and specialization into mesodermal lineages. These data suggest we can obtain MSCs conveniently from different mammals. Their multipotency makes them an invaluable tool for modeling different neurodegenerative diseases or therapeutic applications. 

### 2.2. Isolation and Culture of OE-SCs from Humans

Different biopsy approaches have been established to isolate nasal olfactory MSCs. One non-invasive method developed by Benítez-King et al. involves using a special mini-brush in the nasal epithelium containing neural precursor cells [33] (Figure 1). The cells were cultured in DMEM/F12 supplemented with FBS, L-glutamine, and antibiotics. These cultured cells were still able to specialize into neurons by showing typical cytoskeletal phenotypes such as lamellipodia, filopodia, microspikes, and neurites, suggesting that the cultured neural precursors constitute an excellent model to study the total neurodevelopmental process in the pathogenesis of the disease.

Several other reports have described the biopsy of human nasal mucosae to obtain OE-MSCs [34,35,36,37]. Biopsies were performed on the olfactory mucosae, commonly found in the upper middle turbinate septum or dorsal areas, under local anesthesia. Biopsies were enzymatically dissociated first by incubating the tissues in a solution containing collagenase, then incubated with soya bean trypsin inhibitor and DNase, pipetted, and triturated mechanically through a needle. 

To culture the OE-MSCs, the culture condition can be a serum-free culture medium [34,35] or the DMEM/F12 medium supplemented with FBS [36,37], depending on the specific aims of the studies. For instance, to determine the sphere-forming capabilities of OE-MSCs obtained from humans, culturing the cells in a B27 medium supplemented with N2 medium or in a serum-free defined medium containing epidermal growth factor and basic fibroblast growth factor can give rise to free-floating spheres [34,35]. Serum-free media provides more consistency for experiments between batches and offers researchers more control over culture conditions than the media with serum (Table 2), which could be a good direction for further research. Analysis of surface markers using the flow cytometer indicated that OE-MSCs taken from the nasal mucosa expressed numerous stem cell indicators, such as CD105, CD90, CD73, CD45, and CD34. Moreover, these cells could differentiate into osteocytes and adipocytes [37], indicating their pluripotency. The studies suggested that the isolated OE-MSCs express MSC-specific markers, form spheres, and are clonogenic. When appropriate culture conditions are provided, they can be differentiated into specific tissue cells.

## 3. Differentiation of OE-MSCs into Neural Cells

**Figure 2:**
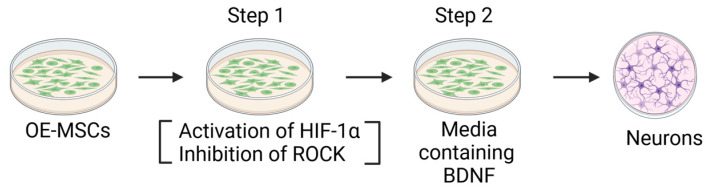
Differentiation of OE-MSCs into neurons.

For cells to differentiate, there should be cell cycle arrest, and neuritogenesis, mediated by the Rho-ROCK signaling system, should occur. Activation of the Rho-ROCK pathway inhibits axon formation, whereas its inhibition induces neurite outgrowth [38]. Also, low oxygen levels aid in neurogenesis from cerebral progenitors by activating the hypoxia-inducible factor-1 (HIF-1α) [39]. HIF-1α is stabilized in normoxic conditions with cobalt chloride (CoCl_2_). Studies also show that BDNF (brain-derived neurotrophic factor) increases when there is a decrease in oxygen concentration in a cell incubator [40]. In order to differentiate OE-MSCs into neurons, a two-step protocol was used in some studies involving HIF-1α stimulation and inhibition of ROCK (Rho-associated kinase) [38]. First, cells were cultured in a neurobasal medium containing B27 fortified with glutamine, FBS, CoCl_2_ (exerting a hypoxic effect on cells to activate HIF-1α), Y-27632 (a ROCK inhibitor), and di-butyryl-cAMP for four days. Second, the same media supplemented with the BDNF, without CoCl_2,_ was used for 3–4 days [39] (Figure 2). This procedure successfully induces neurons, providing reassurance about the effectiveness of the process, as the first step leads to cell cycle arrest in addition to the induction of HIF-1α, and the second helps to induce neuronal growth, differentiation, and survival.

**Figure 3:**
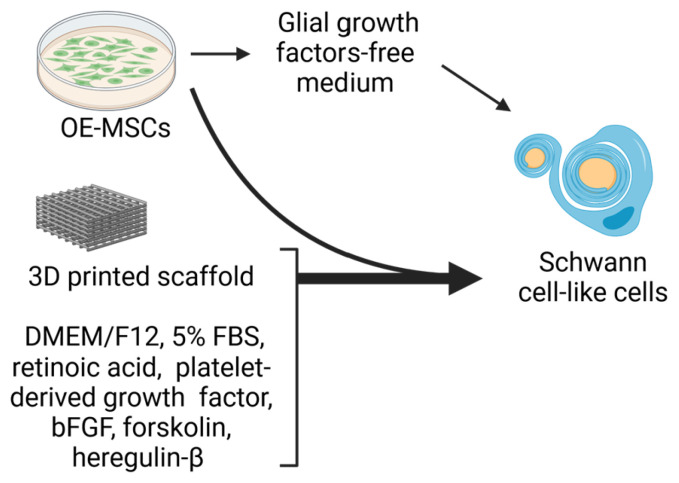
Differentiation of OE-MSCs into Schwann cell-like cells.

In addition to neurons, OE-MSCs can differentiate into other types of neural cells, including spontaneous specialization into Schwann cell-like cells (Figure 3) that express markers of Schwann cells, such as SOX10, p75, S100, GFAP, and MBP, and secrete neurotrophic indicators, such as BDNF and nerve growth factor [41]. When co-cultured with the PC12 cells, a rat phaeochromocytoma cell line that can respond to nerve growth factors and can be induced to specialize into neuronal cells, the co-cultured OE-MSCs showed a neurotrophic effect on PC12 cells by promoting neurite outgrowth to increase neurite length [41]. Moreover, the authors also developed a three-dimensional (3D) cell culture system to enhance the specialization of olfactory epithelial-mesenchymal human stem cells into functional cells exhibiting Schwann cell properties [42] (Figure 3). Results indicated that the 3D scaffold could support the specialization of OE-MSCs into Schwann cell phenotypes to promote neurite outgrowth. Hence, the cells and the culture system have a strong potential as useful models in neural tissue engineering applications. These data suggest that human OE-MSCs have neurotrophic properties and could spontaneously differentiate into Schwann cell-like cells, which may be used to treat peripheral nerve injuries. 

## 4. OE-MSCs Derived from AD Patients Serve as a Model to Study the Disorder 

**Figure 4:**
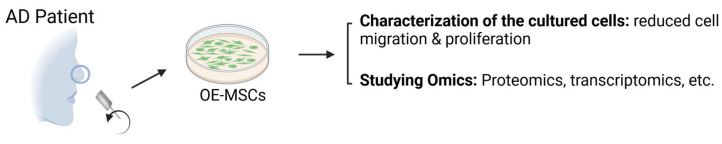
AD patient-derived OE-MSCs, a model to study the disease.

Olfactory dysfunction, an early clinical symptom of AD, could serve as a potential diagnostic indicator of AD severity and progression [43,44]. One way to model AD in cell cultures is to directly reprogram AD patients-derived fibroblasts to form neural stem cells by enhancing the expression of specific transcription factors [45]. However, OE-MSCs emerge as another ideal model to study the pathogenesis of the disease. The potential of OE-MSCs as a model for studying AD pathogenesis is significant. With this model, identifying changes in patient-derived OE-MSCs associated with AD may contribute to the discovery of significant disease biomarkers. This is a significant step forward in understanding AD pathogenesis and identifying biomarkers. To this end, familiar AD patient-derived OE-MSCs from olfactory mucosa biopsies have been isolated and cultured to determine whether any biological properties of the MSCs are associated with AD pathology [46]. Interestingly, it was revealed that AD-derived OE-MSCs showed reduced migration and proliferation compared to those derived from the non-cognitive impaired control individuals (Figure 4). Moreover, the AD subjects with partial or complete loss of smell showed evident atrophy of hippocampal formation, and a large portion of the anosmic AD patients correlated with the ApoE ε4 allele [46].

The study’s findings on the pathogenesis of AD are significant. To understand the pathogenesis of AD, one study compared proteomic alterations linked with Familial Alzheimer’s Disease (FAD) in OE-MSCs obtained from the patients who carry PSEN1 (A431E) mutation with the similar age-matched healthy control people using a liquid chromatography-mass spectrometry approach without label [25] (Figure 4). The authors compared the results between these two (AD patients and healthy patients) and between those patients with pre-symptoms and healthy control cells. Their results revealed an impaired protein expression pattern in an AD stage-dependent manner involving metabolism, vesicular transport, actin cytoskeleton, cell proliferation, and proteostasis pathways, suggesting that OE-MSCs from AD patients may be an ideal model for in vitro studies. Using the same OE-MSC models, transcriptomics analysis of miRNAs showed a marked variation in miRNA expression among various symptomatologic groups and between those with the mutation [47] (Figure 4). Further analysis of the identified miRNA genes of interest indicates that they play a significant role in pathways involved in regulating the pluripotency of stem cells like senescence, cell cycle, and transcription and signaling. This supports the idea that AD patient-derived olfactory MSCs may be a reliable model for investigating the pathogenesis, monitoring disease processes, and identifying novel biomarkers for accurate diagnosis, a positive prospect for the future of AD diagnosis.

## 5. Treating AD with Healthy Individual-Derived OE-MSCs

**Figure 5:**
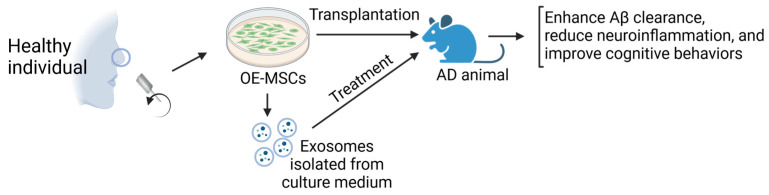
Healthy individual-derived OE-MSCs serve as therapeutics to treat AD.

While AD patient-derived OE-MSCs are a good model for studying the disease, healthy people-derived OE-MSCs can be therapeutic agents due to their beneficial effect on neuronal survival and function. Transplantation of OE-MSCs into the APPswe/PS1dE9 double transgenic AD mice showed an increased amyloid-β (Aβ) clearance, reduced inflammatory response, elevated M2/M1 glial cell ratio, and enhanced learning and memory capabilities compared to the control treatment [48] (Figure 5). Also, healthy OE-MSCs enhanced microglia types’ M1 to M2 conversion in an AD cell model [48]. This study indicates that human OE-MSC transplantation could effectively treat AD. 

In another study, a transplant of human OE-MSCs into an AD rat model either via intranasal spray or intrahippocampal injection showed that intranasal delivery of OE-MSCs was more effective than intrahippocampal injection in improving cognitive deficits in AD mice [49]. OE-MSCs also enhanced BDNF production and reduced Aβ accumulation in AD rats (Figure 5). Therefore, intranasal administration of OE-MSCs may be feasible for treating AD.

Besides OE-MSCs, the secreted products of culturing MSCs, especially the exosomes, are also therapeutic agents. Given the report that the exosomes of the MSCs obtained from the Wharton’s jelly of the human umbilical cord showed marked improvement in spatial memory in AD mice, OE-MSCs derived exosomes may have a similar therapeutic effect on AD [50] (Figure 5). With their small size, low immunogenicity, and high therapeutic efficacy, the MSC-derived exosomes may be advantageous over MSC transplantation [51,52,53].

Moreover, OE-MSCs and their differentiated cells can serve as drug carriers for the delivery of specific drugs through the nasal route because intranasally delivered MSCs or their derived cells can cross the blood-brain barrier into the brain by migrating extracellularly along the olfactory neural pathway with minimal target to other organs [54,55]. Upon entry into the brain, the cells can get to damaged regions in the brain, where the cells not only proliferate and differentiate into neural cells to replace the damaged cells but also release their cargo (either small molecule drugs, neurotrophic factors, or any other therapeutics) to repair the damaged brain. As this is a non-invasive means, it avoids potential adverse effects associated with systemic absorption of drugs. Thus, intranasal administration of MSCs or their derived cells loaded with various therapeutics holds great promise in treating AD and numerous other neurological diseases.

Despite the above-described discoveries, the therapeutic effect of OE-MSCs in AD patients remains unknown. However, according to the website https://clinicaltrials.gov/ accessed on 5 July 2024, over 10 Phase I clinical trials have been conducted to assess the safety and dosage tolerance of different tissue-derived MSCs, such as those found in the blood obtained from the umbilical cord of humans. Based on the limited number of clinical trial studies, single or multiple-repeated delivery of this source of MSCs into the hippocampus, precuneus, or lateral ventricle was practical, relatively safe, and well-tolerated [56,57]. However, the effectiveness of the treatment in AD remains to be determined in advanced phases of clinical trials.

## 6. Future Directions and Challenges

Although prior studies have suggested OE-MSCs as a potentially valuable agent in regenerative therapies for AD, many significant issues remain. One issue is how to standardize the techniques of MSC isolation, as different laboratories used different approaches in isolating and culturing the MSCs, yet there are no definitive markers for the exclusive isolation of OE-MSCs. Also, some currently used MSC markers, such as CD13, CD29, CD44, CD73, CD90, and CD105, fail to distinguish between MSCs and mesenchymal cell cultures that are not stem cells [58]. To solve this issue and identify MSC-specific markers, Wetzig et al. compared MSCs and non-stem cell mesenchymal cell cultures and found three differentially expressed markers, CD24, CD108, and CD40, among which CD24 and CD108 show increased expression in MSCs [58]. In contrast, CD40 shows reduced expression in MSCs. These three molecules may serve as MSC-specific markers. Similarly, through comparison of OE-MSCs and non-stem cell OE cultures, it is likely to identify OE-MSC-specific markers.

In addition to the isolation procedure, the culture, expansion, freezing, and thawing may need to be standardized to ensure the quality standards required for clinical applications. To this end, the Good Manufacturing Practices (GMP)-compliant environment should be considered and developed to meet the clinical use of OE-MSCs. To achieve this goal, Kelly et al. demonstrated that human OM-MSCs could be acquired without adding antibiotics or undergoing enzymatic digestion [59]. The cells thrived in a GMP-compliant culture medium and expressed typical MSC surface markers. The MSCs produced pro-myelinating factors in cultures. However, in vivo work did not show any significant therapeutic effect on myelination [59], suggesting that additional pre-clinical work is needed for better isolation and purification. 

Although prior research has shown OE-MSCs differentiate into neurons and glia, how to selectively differentiate OE-MSCs into functionally specific types of neural cells or specific neural precursor cells remains an issue. Another challenge is that OE-MSCs cannot be cultured in vitro for long due to their spontaneous differentiation, aging, and senescence. Moreover, a sufficient number of MSCs are needed to achieve the therapeutic effect, which requires long-term in vitro expansion of the MSCs. However, this will eventually result in aging or cellular senescence issues, reducing functionality and impaired therapeutic capacity due to the MSCs secreting degenerative factors. To overcome the problem, a variety of MSC rejuvenation strategies have been proposed, including epigenetic reprogramming of MSCs via non-coding RNAs, the small molecule compounds that can reverse MSC senescence, pre-treatment of MSCs with growth factors, transcription factors, or paracrine factors, or physiological preconditioning [60,61,62,63,64]. Currently, to produce large quantities of OE-MSCs in culture remains a challenge. One solution is to utilize a three-dimensional large-scale hypoxic production system that uses GMP-grade for MSCs, which promotes a steady MSC cluster distribution [65]. This procedure also enhances immunosuppression and anti-aging properties, providing uniform cell subpopulations with good-quality MSCs that serve as reasonable grounds for the clinical application of stem cell therapies. However, to better employ OE-MSCs in treating AD, future research should consider designing effective strategies to overcome these problems.

## 7. Summary

Owing to their beneficial characteristics such as multipotency, high proliferation rate, facile accessibility, ability to differentiate into neural cells, low propensity for rejection following transplantation, and no major ethical concerns, OE-MSCs are promising in regenerative medicine for studying and treating AD. Over the past two decades, the methods of isolation, culture, and differentiation of OE-MSCs into specific neural cells have been established, with which OE-MSCs are increasingly utilized in various pre-clinical and clinical studies. While AD patients-derived OE-MSCs serve as a reliable model for studying AD pathogenesis and identifying biomarkers, healthy individual-derived OE-MSCs have significant therapeutic effects that could alleviate AD pathology and improve cognitive functions. Despite being promising, several issues still need to be solved, such as standardizing the isolation procedure of MSCs and dealing with aging and senescence problems. To better employ OE-MSCs in treating AD, future research should design novel strategies to overcome these problems.

## Figures and Tables

**Table 1:** Comparison of different methods in culturing OE-MSCs.

Methods	Advantages	Disadvantages	References
To directly culture tissue cells for 2–3 days before splitting cultures	Relatively high yield of OE-MSCs	Potential contamination with non-OE-MSCs	[29]
Differential adhesion methods by removal of suspension cells after 8 or 18 h	Relatively high purity of OE-MSCs	Relatively low yield of OE-MSCs	[27,28]

**Table 2:** Comparison of different culture media in culturing OE-MSCs.

Medium	Advantages	Disadvantages	References
Serum-containing medium	Relatively cheap	Less consistency in experiments between batches	[35,36,37,38]
Serum-free medium	More consistency in experiments between batches	Relatively expensive	[35,36]

## Data Availability

Not applicable.
